# Supplementary material for: Are carbon emissions trading and green financial instruments synergistic? -Comprehensive quantitative research based on content analysis
Source: PLoS One. 2024 Mar 7;19(3):e0298601. doi: 10.1371/journal.pone.0298601 (PMC10919601; doi:10.1371/journal.pone.0298601)
Supplement: S1 Data — (ZIP) [file pone.0298601.s001.zip › Data/地级市Z_F工作报告.zip!/╣≤╓▌/░▓╦│╩╨/└┤╫╘╣1⁄2╓┌║┼í╛┬φ┐╦╩2╛▌═°í┐_2009.docx]

各位代表：

现在，我代表市人民政府，向大会作工作报告，请予审议，并请各位政协委员和其他列席人员提出意见。

2008年政府工作回顾

2008年是我市改革发展进程中不寻常、不平凡的一年。一年来，我们在中共安顺市委的坚强领导下，在市人大、市政协的监督、支持和帮助下，高举中国特色社会主义伟大旗帜，以邓小平理论和“三个代表”重要思想为指导，深入贯彻落实科学发展观，认真履行政府职责，团结带领全市各族人民，迎难而上，顽强拼搏，抢抓机遇，锐意进取，战胜了严重的雪凝灾害、洪涝灾害和病虫灾害，积极应对国际金融危机带来的冲击，全市经济社会在克服困难中保持平稳发展势头。

2008年全市生产总值168.99亿元，同比增长8.1%；财政总收入26.42亿元，同比增长10.0%，其中地方财政收入16.13亿元，同比增长7.1%；纳入地方统计的固定资产投资54.92亿元，同比增长6.9%；社会消费品零售总额48.12亿元，同比增长21.8%；城镇居民人均可支配收入12249元，实际增长3.7%；农民人均纯收入2838元，实际增长10.2%。一年来，我们主要抓了以下工作：

一是着力推进经济发展。把基础设施建设作为经济发展的重要推动力量，加快发展的基础条件得到改善。城市发展战略规划、城市总体规划大纲通过省级评审，土地利用总体规划修编工作进展顺利，启动了中心城区路网专项规划编制工作。普定经西秀至紫云高速公路纳入全省高速公路路网规划。二环路建设和中华东路改造等项目有序推进，完成两六路安顺段建设和黄果树大街改造工程，市污水处理厂、垃圾填埋场建成并开始调试运行。灞陵河大桥建设和长昆铁路安顺站选址工作进展顺利。完成农村公路建设1000公里。治理病险水库9座，完成7.6万亩烟水配套工程，新增有效灌溉面积6.59万亩，解决了2.7万户、12.2万人饮水安全问题。

以建设安顺民用航空产业国家高技术产业基地为契机，进一步加大抓工业的力度。航空产业基地发展规划与空间布局规划获省政府批准，基地核心区内的道路、供水、供电等基础设施建设扎实推进。汽车产业实现新突破，莲花竞速轿车成功下线。普定循环经济工业基地规划通过省级评审，西秀工业园区、平坝夏云工业园区初步形成产业集群。能源、装备制造、化工、药品、食品等优势产业不断壮大，白酒产业焕发新的生机。全年实现工业增加值52.87亿元，同比增长6.1%。

把旅游业作为第三产业的龙头来抓，第三产业平稳增长。安顺市旅游业发展总体规划编制工作有序推进。“黄果树”成为我国自然风景区唯一“中国驰名商标”，镇宁夜郎洞景区被评为国家4A级景区。贵州省旅游人才培训基地落户黄果树。完成黄果树旅游集团股份有限公司重组工作，成立了屯堡风景名胜区管理处。成功举办了黄果树瀑布节等旅游节庆活动。全年共接待游客1123万人次，同比增长17.1％；旅游总收入82.97亿元，同比增长54.0％。第三产业实现增加值67.19亿元，同比增长10.1％。

把发展生态畜牧业作为加快农业农村经济结构调整的主攻方向，农业农村经济稳步发展。粮食总产量连续六年创历史新高，达80.43万吨，同比增长1.82%。果蔬、优质油菜、烤烟、茶叶、中药材等产业进一步壮大。畜牧业发展势头良好，畜牧业产值占农林牧渔业总产值的42.5%。编制完成农村人口人均半亩基本口粮田建设规划。新阶段扶贫开发深入推进，全年减少农村贫困人口3.52万人，完成易地扶贫搬迁500户、2000人。完成农村贫困劳动力就业转移培训1.63万人，转移农村劳动力5.91万人。新农村建设稳步推进。全市农林牧渔业总产值54.16亿元，同比增长5.79%。

二是着力加强以民生为重点的社会建设。全面完成雪凝灾害损毁房屋重建工作。完成1219户农村危房改造试点任务。启动了农村危房改造扩大试点工作。首创廉租房租售并举经验，建设廉租房3.21万平方米、经济适用房33.5万平方米。城镇新增就业1.42万人，城镇登记失业率4.2%。帮助返乡农民工实现就业3.2万人。全面落实国家提高城乡低保补助水平和企业退休人员基本养老金政策，城乡社会保障体系和社会救助体系进一步完善。城镇居民基本医疗保险工作全面推进。实现基本养老和失业保险市级统筹。产品质量和食品安全专项整治工作取得阶段性成效。“十件实事”基本完成。

镇宁、关岭自治县顺利通过省政府“两基”复查和“普实”验收。扎实推进农村义务教育经费保障机制改革，全部免除义务教育阶段学生学杂费，“普九”债务化解工作全面启动。完成农村中小学薄弱学校改造工程65所。安顺职业技术学院顺利通过教育部高等职业院校人才培养评估。重大传染病和地方病防治力度不断加大，完成地氟病病区家庭炉灶改良1万户。新型农村合作医疗人均补助标准提高到80元，参合率达93.83%。人口出生率和自然增长率分别下降至12.86‰和6.2‰。文化体制改革迈出重要步伐，组建黔中报业有限公司。成功举办格凸全国攀岩精英挑战赛、全国“铁人”两项赛等一系列重大赛事活动。组队参加贵州省首届农民运动会，取得17块金牌的好成绩。全民健身活动广泛开展。精神文明建设深入推进。老龄、妇女儿童和残疾人事业取得新进步。人事人才工作进一步加强。国防建设和经济建设协调发展。新闻出版、民族宗教、外事、侨务、对台、人防、地方志、红十字、档案、气象等工作取得新成绩。

扎实开展“安全生产百日督查专项行动”，安全生产事故和死亡人数实现“双降”。“平安安顺”创建活动深入推进，人民群众的安全感得到提高。以开展“县委书记大接访”和干部下访为契机，进一步加强信访工作，有效维护了社会稳定。

三是着力加强生态环境建设。完成营造林12.08万亩，新建环城林带1万亩，全市森林覆盖率达到38%。完成农村沼气池建设1.5万户。全面开展石漠化综合治理试点工程。治理水土流失面积52.8平方公里。全市万元GDP能耗同比下降4.41%。重点流域污水排放的监管和污染治理力度加大。城区空气优良率达100%，集中式饮用水源地水质稳定达标，夜郎湖水质达二类水标准。

四是着力推进改革开放。认真总结改革开放30周年、安顺试验区成立20周年和“顶云经验”30周年的实践经验，全面推进各项改革。编制完成了安顺试验区改革发展总体规划。国有企业改革改制工作稳步推进。集体林权制度改革全面展开。平坝县进入全省经济强县行列。安顺市商业银行获准筹建。组建了市金融服务办公室。百灵制药上市工作取得新进展。非公经济发展加快，市县属非公有制工业企业实现增加值18.78亿元，同比增长19.6%，占市县属工业增加值的88.5%。引进了投资4.9亿元的贵州云马厂民用航空零部件转包生产、注册资本1亿元的贵州中航通用飞机有限责任公司等项目。河南永煤集团、江苏雨润集团等一批大企业入驻安顺。成功开展走进多种经济成份共生繁荣改革试验区——企业家安顺行活动。“九顺合作”取得新进展。在上海、广东两地设立了政府对外联络处。与长三角、珠三角等发达地区和对口帮扶城市青岛、友好城市苏州的联系进一步加强。

五是着力加强政府自身建设。深入开展学习实践科学发展观活动，进一步明晰了科学发展的思路，增强了科学发展的自觉性和坚定性。坚持民主集中制原则，不断完善政府议事决策机制。自觉接受人大的法律监督、工作监督和政协的民主监督，认真办理各级人大代表建议111件、政协提案211件，办复率100%。普法依法治理工作有序推进。行政执法责任制和问责制落实力度不断加大。应急管理工作进一步加强，预防处置突发公共事件的能力得到提高。全面贯彻《政府信息公开条例》，切实推进行政权力公开透明运行。完成政府办公自动化一、二期工程。政府系统党风廉政建设进一步加强。

各位代表，过去的一年，我们接连经历了一些难以预料、历史罕见的重大挑战和考验。面对种种困难，我们牢固树立全局意识，大局意识，围绕“保民生、保发展、保稳定”，积极开展抗灾救灾和灾后恢复重建工作，夺取了抗灾救灾工作的重大胜利。积极支援汶川地震灾区抗震救灾和灾后重建工作，累计向灾区捐赠款物2465万元。我们积极抢抓国家扩大内需促进经济发展的重大机遇，争取到中央新增1000亿元拉动内需投资项目174个、落实中央补助资金2.72亿元，启动了一批多年想上而没能上的项目。

2008年取得的成绩难能可贵、来之不易。这是省委、省政府和市委正确领导的结果，凝聚着全市人民的心血和汗水，得益于市人大、市政协的监督和支持。在此，我代表市人民政府，向全市工人、农民、知识分子、干部，向各民主党派、工商联、无党派人士、人民团体，向驻安人民解放军指战员、武警部队官兵、公安政法干警，向所有关心和支持我市改革开放和现代化建设的各界人士，表示衷心的感谢，并致以崇高的敬意！

在肯定成绩的同时，我们也清醒地认识到，在经济社会发展和政府工作中还存在一些困难和问题。主要表现在：一是经济总量小、人均水平低、财力弱的状况还未得到根本改变，加之受国际国内急剧变化的经济形势影响，进一步加快发展的任务艰巨。二是产业结构不尽合理，工业化水平低，工业园区建设滞后；旅游产业化程度低，旅游资源优势没有充分转化为经济优势；农业基础薄弱，保持农民持续增收难度大。三是城镇化水平不高，城市建设滞后，中心城市辐射带动作用不强。四是尽管纳入地方统计的固定资产投资实现增长，但由于跨区项目减少，全社会固定资产投资仍呈下滑趋势，投资拉动经济增长的动力不足。五是发展环境尤其是软环境建设滞后，与进一步加快发展的要求还不相适应。六是部分群众生活还比较困难，就业压力大，在征地、拆迁、移民、企业改革改制等工作中影响社会稳定的因素仍然较多，安全生产形势不容乐观。七是一些职能部门的服务意识和执行力、落实力不强，影响了全市发展大局。对这些问题，我们将高度重视，积极采取有效措施，切实加以解决，并诚恳地接受各位代表、委员和社会各界的监督与批评，努力把政府工作做得更好，向全市人民交出一份满意的答卷,绝不辜负全市各族人民的期望和重托！

2009年政府工作主要任务

2009年是新旧矛盾相互交织、挑战和机遇并存的一年。国际金融危机仍在蔓延，对我国一些行业的影响还在扩展和加深，将会给我们的发展带来更大的困难、产生更大的影响。对此，我们要保持清醒认识。同时，也要看到我们应对危机、加快发展的积极因素和有利条件：一是中央全面实施促进经济平稳较快增长的一揽子计划，西部大开发战略的深入实施，为我们加快发展提供了强有力的支持。二是经过改革开放30年尤其是试验区创建20年的实践和探索，我们形成和积累了一些好的制度机制和经验，为进一步加快发展打下了坚实的基础。三是目前急剧变化的国内外市场需求形势，虽然给企业发展带来了较大压力，但也为我们淘汰落后生产能力，承接发达地区产业转移，加快推进产业结构调整创造了机遇。四是我市的发展思路进一步明晰，全市上下盼发展、思发展、谋发展的愿望强烈，同时我市正处于工业化、城镇化加快发展阶段，加速发展的内在动力强。我们面临的机遇大于挑战，有利因素大于不利因素。我们要进一步增强信心，振奋精神，把困难估计得更充分一些，把措施准备得更周密一些，努力变压力为动力、化挑战为机遇，聚精会神搞建设，一心一意谋发展，扎扎实实做好各项工作。

2009年政府工作的总体要求是：高举中国特色社会主义伟大旗帜，以邓小平理论和“三个代表”重要思想为指导，深入贯彻落实科学发展观，继续解放思想，坚持能快则快，按照党中央、国务院，省委、省政府和市委的一系列决策部署和工作要求，以抓项目、强基础、促发展为首要任务，以保障改善民生、促进社会和谐为出发点和落脚点，以加快转变发展方式和优化产业结构为主攻方向，以深化改革扩大开放为动力，着力推进工业化、城镇化、旅游产业化、农业现代化和优化发展环境“四化一优”工作，努力实现经济社会又好又快发展。

2009年经济社会发展的主要预期目标是：生产总值增长10%以上；纳入地方统计的固定资产投资增长18%以上；社会消费品零售总额增长15%以上；财政一般预算收入增长10%以上；城镇居民人均可支配收入实际增长8%以上；农民人均纯收入实际增长8%以上；城镇登记失业率控制在4.5%以内；人口出生率和自然增长率分别控制在13‰和6.7‰以内；单位生产总值能耗比上年降低4.3%以上，主要污染物排放减少达到排放要求。

围绕上述总体要求和主要预期目标，今年我们将重点抓好以下十个方面的工作。

（一）在抓项目建设上下大力，促进全市经济平稳较快增长

扎实推进以交通水利为重点的基础设施项目建设。积极配合做好黄织铁路、灞陵河大桥、厦蓉高速公路平坝段、惠水经紫云至贞丰高速公路、六枝至大山高速公路的建设工作和长昆铁路前期工作。开工建设普定至西秀高速公路。尽快实施煤矿运输公路和铁路联运项目。抓紧完成黄果树机场道面改造工程。完成163公里通乡油路和26.1公里通村油路建设，力争年内实现所有乡镇通油路或水泥路，所有行政村通公路。配合做好黔中水利枢纽一期工程，确保工程顺利实施。抓好油菜河水库、鲁嘎水库“滋黔”工程建设，做好安西灌区续建配套与节水改造工程。加快紫云黄家湾水利枢纽项目前期工作，争取早日立项。继续配合做好光照、董箐、马马崖等重点工程建设。完成格八水库和小（一）型病险水库治理任务，做好小（二）型病险水库治理前期工作。改造中低产田土2.69万亩，整理复垦开发土地4.25万亩。积极配合做好城乡电网建设和改造工程，提高城区电缆入地率，完善供电网络，加快实现县县有220千伏变电站，增强供电保障能力。抓好信息基础设施建设，加大“数字安顺”建设力度，推进“三网融合”。推动第三代移动通信网络建设。开通中国移动多媒体数字广播。

切实提高项目工作水平。牢固树立抓项目就是抓发展、抓民生、抓就业、抓财源、抓跨越的思想，大力推进项目建设，把项目工作作为经济工作的“牛鼻子”，作为有效应对金融危机的重要抓手。加大项目前期工作力度，建立项目前期工作责任制，力求在项目前期工作上取得突破性进展。加强项目库建设，坚持一手抓基础设施项目，一手抓产业发展项目，谋划更多关系全局、能够带动全市经济社会发展的大项目、好项目，切实增强项目申报的说服力、对投资者的吸引力、对经济社会发展的带动力。根据国家和省扩大内需的政策措施和产业振兴计划，充分利用安顺试验区政策优势，千方百计争取项目。结合我市的资源优势和产业特色，面向市场，千方百计引进项目。充分利用财政资金、社会投资、金融手段等多种形式，千方百计解决项目资金短缺的问题。

坚持能早则早、能快则快的原则，把落实好中央新增投资项目和重点项目建设作为一项重大的政治任务，集中力量攻坚克难，强力推进项目建设。按照提前介入、联合审查、特事特办、限时办结的要求，切实解决项目建设审批难、供地难、融资难、拆迁难的问题，确保项目早开工、早竣工、早投产、早见效。创新项目落实工作机制，进一步强化项目领导机制、服务机制、考核机制、问责机制，全面落实重大项目市级领导牵头督促推进制度，努力实现项目建设“低成本、快回报、高诚信、优服务、零障碍”。

（二）在抓工业发展上求突破，全力推进新型工业化进程

以建设航空产业基地为契机加快发展现代装备制造业。尽快编制完成基地相关控制性详规。加快基地基础设施建设，完成基地内的主干道路建设，推进城市道路建设向基地延伸，促进城市建设与基地建设同步发展。将国家和省安排在我市实施的供电项目向基地倾斜，加快完善供电设施。抓好给排水工程建设，配套建设污水处理厂和污水收集系统。以配合推进30座级飞机生产为重点，加大相关项目引进工作力度，增强产业配套能力。发挥在安军工企业技术、设备及人才优势，开发市场潜力大、附加值高的产品，做大做强农业机械、医疗器械、石油钻井机具、大型成套设备零部件、精密轴承、铁路机车配件等产品。大力发展汽车产业，支持莲花轿车、云马客车、特种车及汽车零部件生产。

进一步培育壮大以能源、化工和原材料为主的支柱产业。全面实施我市工业振兴计划。抓紧做好全市煤炭资源详查和精查工作，为电力工业和煤炭产业发展夯实基础。把骨干矿井建设作为发展煤炭产业的关键来抓，规划建设一批大中型骨干矿井，促进煤炭产业从粗放型向集约型转变，尽快实现大煤保大电。抓好大矿帮带小矿工作。加快推进安顺电厂三期项目前期工作，力争早日开工建设。以煤化工、煤电化、煤电铝一体化为重点，加快实施一批重大产业项目，促进原材料工业资源整合，延长产业链，实现产业内部利益最大化。推进以新型环保建材为主的建材工业发展。

加快改造提升传统特色优势产业。支持以苗药为重点的拥有自主知识产权的药品开发和技术创新，抓好百灵集团上市的指导和帮扶，提升我市民族药业的市场竞争力。以肉制品、辣椒制品、调味品和山药食品等为重点，大力发展食品工业。整合资源，做强品牌，进一步培育壮大茶产业。完成安酒集团政策性破产和重组工作，重塑安酒品牌形象，支持平坝酒、黄果树酒、夜郎酒进一步发展，振兴白酒产业。加大旅游商品研发力度，推进蜡染、民族刺绣、地戏脸谱、古生物仿制品、航空模型和波波糖等产品规模发展，形成富有安顺地方特色的旅游商品系列。

以园区为载体加快推进产业聚集。树立经济建设以工业突破为主、工业突破以招商引资为主、招商引资以项目进园区为主的思想，紧紧依托我市规划建设的四个工业园区，加大产业项目进园区力度，提高产业集中度和产业配套功能，减少发展成本，扩大规模效益。抓住发达地区产业结构调整转移的机遇，结合园区的特点和定位，积极做好承接产业转移的工作，打造工业经济新的增长点。建立和完善工业园区市县两级共建共享机制。对进入园区产业项目的洽谈、签约、开工、建设等实行全程跟踪服务，确保引进一个，落地一个，建成一个，见效一个。

加大对工业企业的扶持力度。认真落实国家对企业的各项税收减免政策和实施增值税转型改革政策，积极争取省贷款贴息和无偿资助等资金，把服务企业作为推动产业发展的头等大事抓好抓实，帮助企业解决资金、产销衔接、技术创新等方面的问题。对骨干企业实行“VIP”服务制度，在用地、电力、资金等方面进行重点支持。进一步加强对天马轴承、金叶科技、宏盛化工、黄果树铝业、红星发展、关岭永诚、宏泰化工、超宇水泥和昌兴水泥等企业的协调服务工作，促进企业尽快做大做强。

增强科技创新能力。以引进消化吸收再创新为重点，加强产学研结合，加快构建区域科技创新和科技合作交流体系，推进科技成果向现实生产力转化。依托民用航空产业基地建设，积极争取国家和省重大科技专项在我市实施。开工建设航空产业孵化中心。加强知识产权创造、运用和保护能力建设。充分发挥企业技术创新的主体地位，加大工业项目技改力度，引导企业加快结构调整步伐，提高产品的市场竞争力。

（三）在抓城市建设上强举措，全力推进城镇化进程

加强以路网为重点的城市基础设施建设。按照“南北贯通，东西拓展，完善功能，建设新区”的思路，加大城市基础设施建设。完成龙青路、南航路建设和中华东路续建工程，开工建设体育路、安普路一期、中华西路、星火路、西秀工业园区大道等工程，加快二环路、机场路等建设步伐，进一步拓展和完善中心城区道路骨架网络。加快黄果树大街沿线区域的开发建设，形成新的城市景观大道。依托虹山湖、娄家湖等资源，打造独具特色的城市精品区。加大历史文化街区的保护和开发力度，丰富和增强城市历史文化底蕴，提升城市品位。

增强城市服务功能。完成城市公共交通枢纽总站主体工程、城区站台和城市管道燃气接收站建设。完成中心城区照明设施、城区道路给排水设施维修改造和城区道路绿化工程。完成市殡仪服务中心主体工程80%。抓好贯城河治理工程、城区供水管网二期改扩建和城区污水管网改扩建工程。加快市人民医院搬迁、市二中新校区建设。启动市生活垃圾收运系统和市医疗垃圾废物集中处理工程建设。

提升城市规划管理水平。按照建设百万人口大城市的定位，完成城市总体规划、土地利用总体规划修编，编制完成城市路网规划和城市公共交通专项规划。编制完善县区城镇体系规划和小城镇规划。进一步理顺市区两级城市建设管理体制，充分调动两城区在城市建设管理中的积极性和主动性。完成城区15条小街小巷改造工程。加强市政设施管理，为市民创造安全、便利、舒适的生活环境。

加快城镇化进程。深化户籍制度改革，探索把在城市中有相对固定住所和相对固定工作的本市籍进城务工人员转变为城市居民。以有条件的建制镇和村为基础，加快“镇改办”、“村改居”步伐，实现农村居民身份到城镇居民身份的整体转变。加强“城中村”改造，启动管元村改造和龙井新村、西兴新村、大营新村建设。支持各县区加快县城和具有一定产业支撑、富有地方特色、区位优势明显的小城镇建设，增强其承接中心城市功能、带动农村区域经济社会发展的能力。

（四）在提高旅游产业化水平上下功夫，加快发展第三产业

加大旅游资源整合力度。完成安顺市旅游业发展总体规划编制工作，做好省级以上风景名胜区控制性详规编制工作。围绕构建泛黄果树大旅游圈，加大旅游资源的整合、包装力度，提升旅游产品档次，促进观光游向休闲度假游转变，加快资源优势向经济优势转化。抓好自然风光游、民族风情游、历史文化游、红色旅游、乡村旅游发展，支持以天龙、九溪、本寨、石头寨、木城河、讲义村等为重点的一批乡村旅游产品深入发展。精心设计、合理编排，重点推出一批二日游、三日游精品旅游线路。做好屯堡风景区、关岭花江大峡谷风景区申报国家级风景名胜区工作。推进旅游与会展、体育、科考等相结合，丰富和拓展旅游内涵。

增强旅游接待服务能力。按照国际化、精品化、多元化、现代化、人性化的要求，完善景区的基础设施和配套服务设施。抓好黄果树半边街搬迁和黄果树新城建设。抓紧规划建设一批等级较高的旅游公路和景区连接线，提高进入景区的通达深度和便捷度、舒适度。加快规划建设旅游商品一条街、特色小吃一条街、旅游车站和游客服务中心。着力加强酒店、旅行社等旅游服务设施建设。

深化旅游管理体制改革。进一步完善黄果树旅游集团股份公司现代企业制度，加快上市步伐。理顺龙宫风景区管理体制。支持组建屯堡文化旅游股份公司。出台加快我市旅行社和酒店业发展的政策措施，建立景区和酒店联动互惠机制，推出景区门票“惠民卡”。按照宣传、文化、旅游、体育“四位一体”的思路，开展旅游整体宣传促销，用市场化的办法举办黄果树瀑布节等旅游节庆活动，积极拓展客源市场，提高安顺知名度和美誉度。依托贵州省黄果树旅游人才培训基地，加大旅游从业人员培训力度，提高旅游从业人员综合素质。加强旅游市场监管，营造健康、有序、规范的旅游市场环境。

大力发展第三产业。认真贯彻落实鼓励支持服务业发展的各项政策，不断优化服务业发展环境。加快发展商贸餐饮、文化健身、社区服务等面向民生的服务业，大力发展交通运输、现代物流、金融保险、信息咨询、科技服务等面向生产的服务业。全面落实我市促进房地产业健康发展的各项政策措施，稳定房地产投资和消费预期。

（五）在促进农民持续增收上见实效，推进农村改革发展

全面落实各项强农惠农政策。认真落实好种粮补贴、良种补贴、农机具购置补贴、农资综合补贴，将油菜纳入良种补贴范围，确保各项涉农补贴资金一分不少地及时发放到农民手中。取消病险水库除险加固、生态建设、农村饮水安全、大中型灌区配套改造等公益性建设项目的县及县以下配套资金。加大财政对“三农”的投入，完善支农资金稳定增长机制。开展以县为主的财政支农资金整合试点工作，提高资金使用效益。

加快农业产业结构调整。高度重视粮食安全，坚持最严格的耕地保护制度和最严格的节约用地制度，稳定粮食播种面积，努力确保粮食产量达到78万吨以上。严格执行国家粮食收购质价政策，完善粮食风险基金和地方粮食储备制度。巩固油菜、烤烟等传统优势产业，新增和改造茶园3万亩，种植蔬菜40万亩、中药材1万亩。加快发展生态畜牧业，实现畜牧业产值占农林牧渔业总产值的比重增加1个百分点以上。加快“一乡一特，一村一品”向“多乡一特、多村一品”拓展，建设一批优质农产品生产基地。大力推进农业产业化经营，加大扶持龙头企业的力度，培育特色农产品知名品牌。做好农产品注册商标、质量认证和地理标志申请保护工作。积极推行农业标准化生产，建立健全农产品质量监管机制。大力支持和引导农民专业合作社、农民专业经济组织、农民经纪人队伍加快发展，努力提高农民的组织化程度。

扎实做好扶贫开发和新农村建设工作。认真执行国家新的扶贫标准，对农村低收入人口全面实施扶贫政策。坚持先易后难、样板先行，以基础设施建设、培育特色优势产业和提高人口素质为重点，深入实施开发式扶贫。减少农村贫困人口3.86万人，完成易地扶贫搬迁1000人。解决2.5万户、11万人饮水困难和饮水安全问题。做好水利水电工程移民搬迁安置和后期扶持工作，确保移民生活水平不断提高。扎实推进新农村建设，抓好16个示范村建设工作，充分发挥示范带动作用。加强村寨规划整治，建设一批富有地方建筑景观特色、布局合理、设施配套、环境整洁、融合现代文明的新村寨。

积极推进农村各项改革。保持农村现有土地承包关系长久不变，赋予农民包括离乡农民工更加充分而有保障的土地承包经营权。坚持依法、自愿、有偿的原则，开展农村土地流转试点工作，发展适度规模经营。完成集体林权制度主体改革，推进配套改革。充分发挥金融机构支农作用，着力解决农村资金短缺问题。继续推进农村综合改革。健全农村民主管理制度。

（六）在建设生态文明上创优势，构建资源节约型和环境友好型社会

加强生态环境建设。坚持生物措施和工程措施并举，全面推进石漠化综合治理试点工作。治理水土流失面积100平方公里。加快推进天然林保护、珠江防护林工程和退耕还林工程建设，完成营造林14万亩以上，确保我市森林覆盖率提高1个百分点。加强重大地质灾害区和已开采矿区的生态恢复和治理，依法督促企业足额缴存并切实用好矿山治理恢复保证金。完成农村沼气池建设2万户，加大“改厨、改厕、改圈”工程建设力度，改善农村生产生活环境。

加大节能减排和环境保护力度。坚持从开发和建设的源头控制新的环境污染和生态破坏，降低能源消耗，削减污染物排放总量。全面落实节能减排目标任务，突出抓好重点行业、重点企业的节能管理和技术改造。严格环境准入，规范审批程序，严格建设项目“三同时”制度。优先保障科技含量高、资源消耗少、税收贡献大的项目，确保优质资源向优质载体集聚。引导和支持企业调整产品结构，降低能耗和生产成本，带动行业提高节能降耗水平。完成各县区污水处理厂、垃圾填埋场建设和伍家关垃圾填埋场“封场”工程。制定农村环境保护措施，防止农业面源污染。加强环境监测体系建设，强化对大气污染、城市噪音污染、水污染等的综合治理，强化集中式饮用水源地环境保护，让全市人民喝上干净的水，呼吸到新鲜的空气。

大力发展循环经济。积极推广和开发节约、替代、循环利用资源的先进技术，提高资源综合利用率。以普定循环经济工业基地为载体，加快大宗工业废弃物规模化综合利用项目建设，积极推动循环经济和环保产业项目的发展。大力推进“三废”综合利用，实施煤层气、煤矸石等资源综合利用循环经济项目，鼓励瓦斯抽采及综合利用。坚决淘汰效益差、污染重、不具备市场竞争优势的企业。

（七）在改善保障民生上动真情，努力提高人民群众生活水平

千方百计扩大就业。实施更加积极的就业和再就业政策，充分调动企业、劳动者和社会各方面力量，全力保持就业形势稳定。认真落实国家鼓励高校毕业生到基层就业的各项政策措施。做好公共就业服务工作，扩大普惠制就业培训范围。加大就业援助力度，开发公益性岗位，做好零就业家庭等困难群体就业帮扶工作。大力支持自主创业、自谋职业，促进以创业带动就业。全年完成城镇就业1.25万人（其中下岗失业人员再就业4300人，就业困难人员就业2100人）。加强有组织的劳务输出，引导农民工有序流动。组织农民工参与新农村和拉动内需相关项目建设，鼓励和支持返乡农民工创业。加强劳动争议调解、仲裁和监察执法，切实维护劳动者合法权益。

完善社会保障体系。扩大社会保险覆盖面，实现新被征地农民养老保险全覆盖，实现城镇居民基本医疗保险全覆盖。重点做好非公有制经济从业人员、农民工、灵活就业人员和自由职业者参保工作。强化社会保障基金监管。健全社会救助体系，加快城乡低保信息网络建设，加强动态管理。全面落实农村五保供养政策，确保供养水平达到当地村民平均生活水平。完善城乡医疗救助制度，缓解困难群众看病难问题。建立健全临时救助制度，帮助低保边缘群体、低收入群体解决特殊困难。健全灾害救助制度，提高灾害救助能力。加快社会福利、慈善事业发展。

进一步完善住房保障体系。扎实做好农村危房改造扩大试点工作，确保在4月底前保质保量完成2815户试点任务。全面启动农村危房改造工作，真正把农村危房改造工程办成人民群众满意的实事好事，推动我市农村群众生活质量有一个明显提高，农村面貌有一个明显改善。坚持租售并举、以售为主的方式，完成廉租房建设8.2万平方米，新增租赁补贴1750户。规范经济适用房建设。强化住房公积金使用管理，充分发挥住房公积金制度在住房保障体系中的作用。

努力增加城乡居民收入。完善企业职工工资正常增长和支付机制，推进事业单位工资改革。将工资保证金制度实施范围由建设领域扩大到交通和水利领域，进一步做好预防和解决企业工资拖欠工作。全面落实中央提高城市和农村低保补助水平政策。继续提高企业退休人员基本养老金水平。切实抓好中央优抚对象、建国前老党员和抗日战争、解放战争时期参加革命的离休干部相关补助政策的落实。

积极扩大消费需求。把扩大消费与完善收入分配政策、发展服务业、扩大就业结合起来，积极鼓励居民消费。认真落实国家扩大消费需求的各项政策措施，加强城乡消费设施和服务体系建设，优化消费环境。做好“家电下乡”工作，继续推进“万村千乡市场”建设，积极开拓农村消费市场。

切实维护社会和谐稳定。狠抓安全生产各项责任制的落实，强化煤矿和非煤矿山、交通运输等重点领域的安全监管，坚决关闭不符合安全生产条件的企业，坚决遏制重特大事故发生，实现安全事故起数和死亡人数“双降”。深入开展食品药品安全专项整治，加强对生产流通全过程的监管，加大重点产品抽查力度，坚决杜绝不合格产品进入市场。加强和改进信访工作，重视群众的合理诉求，认真开展矛盾纠纷排查化解工作，切实解决土地征用、房屋拆迁、企业改制、库区移民和环境污染等方面损害群众利益的突出问题。深入开展“平安安顺”创建活动，继续开展以“打黑除恶”为重点的严打专项整治行动，加大社会治安综合治理力度，切实保障人民群众生命财产安全。积极做好防灾减灾工作。高度重视隐蔽战线工作，切实维护国家安全。认真抓好市委、市政府确定的“十件实事”。

（八）在基本公共服务上保公平，努力构建和谐社会

坚持教育优先发展。巩固提高“两基”攻坚成果，确保“两基”通过“国检”。完成农村“普九”债务化解任务。完善义务教育经费保障机制，确保农村中小学公用经费落到实处。继续做好对农村义务教育阶段学生免费提供教科书工作。落实农村贫困家庭寄宿生生活补助政策，完善贫困学生资助体系。实施中小学校舍安全工程，推进农村中小学标准化建设。完成“中西部农村义务教育阶段薄弱学校改造工程”51所。着力解决进城务工人员子女接受义务教育和农村留守儿童教育管理问题。稳定扩大普通高中和中等职业教育招生规模。支持安顺学院和安顺职业技术学院加快发展，提高高等教育质量。进一步加强民族教育、学前教育、特殊教育，大力发展远程教育和继续教育，鼓励和规范民办教育发展。深入推进素质教育，促进学生全面发展。加强教师队伍建设，健全激励机制，全面落实义务教育阶段教师绩效工资制度。认真落实省政府《关于加强全民科学素质工作的意见》，努力提高全民科学素质。

加强医疗卫生建设。完善公共卫生经费保障机制，落实人均公共卫生服务经费标准。巩固和完善新型农村合作医疗制度，使广大参合农民得到更大实惠。完成21个乡镇卫生院建设工作，新建、改扩建9个乡镇计生服务站，增强农村医疗卫生服务能力。加强城市社区卫生服务网络建设，加快构建以社区卫生服务为基础的新型城市卫生服务体系。强化医疗服务和医药价格监管，维护患者利益。高度重视免疫规划工作，抓好重大传染病和地方病防治，全面完成地氟病病区家庭炉灶改良5万户。加强人口和计划生育工作，继续稳定和降低生育水平，综合治理出生人口性别比，开展出生缺陷干预，提高出生人口素质。

大力发展文化体育等社会事业。加强文化基础设施建设，完成县级文化信息资源共享工程建设和22个乡镇综合文化站设施设备的配置，新建“农家书屋”270个。稳步推进文化事业单位体制改革。加强文化市场监管。做好第三次全国文物普查工作。继续实施农村电影放映工程。加快推进20户以上已通电自然村“村村通”广播电视工程。完成县城有线电视网络升级改造。加强体育场所的建设改造和维护，完成74个农民体育健身工程。深入开展全民健身活动，提高竞技体育水平。进一步做好人事人才工作。继续做好新闻出版、民族宗教、外事、侨务、对台、人防、地方志、老龄、妇女儿童、红十字、残疾人、档案、气象等工作。

加强社会主义精神文明建设。以创建文明城市为统领，广泛开展“迎国庆讲文明树新风”、“整脏治乱”专项行动，深入开展“与文明同行、满意在安顺”、文明单位、文明村镇等群众性精神文明创建活动。加强和谐文化建设，开展和谐社区等文明创建活动。大力宏扬“黔中文化”，实施文艺精品工程，着力推出一批有档次、有影响的文艺作品。继续开展“三下乡”活动和“四进社区”服务。深入开展“双拥”工作，争创省级、国家级双拥模范城（县）。加强国防教育，健全国防动员体系。支持人民解放军、民兵预备役和武装警察部队建设。

（九）在深化改革扩大开放上出硬招，激发贵州加快发展经济特区的内在活力

进一步深化改革。按照“超前探索、先行试验、封闭运行、大胆创新”的要求，进一步解放思想，破除影响和制约加快发展的一切障碍，构建有利于科学发展的新体制、新机制，努力把安顺试验区建设成为贵州加快发展的经济特区。大力支持非公有制经济加快发展，全面贯彻落实并不断创新非公有制经济加快发展的各项政策措施，做大做强非公有制经济，着力提高非公有制经济在全市生产总值中的比重。全面实施中小企业成长工程，组建中小企业担保公司，完善中小企业信用担保体系，拓宽融资渠道，培育发展一批影响力大、带动效应明显的龙头企业。基本完成国有企业改制任务，鼓励优强企业兼并重组濒临破产企业。加强国有资产监管，实现国有资产保值增值。认真落实国家推进成品油和天然气价格、医药卫生体制等改革的政策措施。依法加强税收征管，强化财政预算刚性，规范财政资金管理，优化支出结构，提高资金使用效益，集中财力办大事。研究出台我市的分税制改革试行办法。完成安顺市商业银行的筹建挂牌工作。搭建交通建设投融资平台。充分发挥市金融服务办公室的作用，深化政银企合作，切实抓好政府与金融机构合作协议的落实。探索发展村镇银行、小额贷款公司等新型金融组织。按照“市区联动、以区为主”的原则，建立科学合理的市区两级土地收储体制机制。加大闲置土地清理和处置力度，防止“屯地、圈地”行为。积极稳妥推进政府机构改革，全面实行事业单位岗位设置管理，深入推进行政审批制度改革。

进一步提高对外开放水平。把招商引资作为扩大开放的重要抓手，明确各级各部门招商引资工作责任，全面完成招商引资目标任务。完善落实招商引资鼓励、奖励、考核等政策措施，调动社会各界参与招商的积极性。充分利用网络优势，搭建招商引资网络平台。发挥政府驻外办事机构、商会、行业协会和中介组织在招商引资、对外经济协作中的“窗口”和“桥梁”作用，积极推动“以商引商”。充分依托我市优势资源，积极引导、鼓励和支持市内外各类投资者进行投资，进一步壮大投资规模。优化引资结构，坚持招商与选商相结合，切实做到护我环境、惜我资源、利我地方、惠我群众。坚决消除影响发展的“绊脚石”、“中梗阻”和“拦路虎”，大力营造廉洁务实的政务环境、优质高效的服务环境、公开公平的市场环境和诚实守信的信用环境，增强投资环境的竞争力和吸引力。对招商引资重点项目实行“保姆式”服务，让投资者放心投资、安心发展。坚持“走出去”与“引进来”相结合，积极扩大与珠三角、长三角、西南六省区市，以及对口帮扶城市、友好城市的交流与合作。以更加积极主动的姿态参与省内区域合作。

（十）在加强政府自身建设上求实绩，切实提高服务科学发展的能力

加强制度建设，切实提高执行力和公信力。进一步建立健全抓落实的工作制度，让政府系统各级干部有“想抓”的意识、“能抓”的措施、“善抓”的本领、“敢抓”的勇气，创造性地开展工作，不断开创工作新局面。把抓落实作为衡量政府系统各级领导干部思想上是否讲政治、工作上是否有能力的试金石。进一步落实重大工作调度会议制度、重点工程定期督查通报制度，坚决克服重安排、轻落实的倾向，对不执行、虚执行和乱执行的现象实行严格的问责制，确保政令畅通。以新一轮政府机构改革为契机，以转变政府职能为核心，调整和优化政府组织结构与职责分工，规范行政权力。大力推进政务、村务、厂务公开和公共企事业单位办事公开，完善政府新闻发布和信息公布制度，提高工作透明度。

加强民主法制建设，切实提高依法行政的能力和水平。自觉接受同级人大及其常委会的法律监督、工作监督和同级政协的民主监督，认真办理人大代表的议案、建议、批评、意见和政协提案。自觉接受人民法院和人民检察院的司法监督。认真听取民主党派、工商联、无党派人士的意见和建议，充分发挥工会、共青团、妇联等人民团体的桥梁纽带作用，接受新闻舆论和人民群众对政府机关及其工作人员的监督，支持监察、审计部门依法独立履行监督职责。健全基层群众自治机制，扩大基层群众自治范围，完善基层民主管理制度，保障人民群众依法直接行使民主权利、管理基层公共事务和公共事业。认真贯彻落实《国务院关于加强市县政府依法行政的决定》。完善群众参与、专家论证和集体决策相结合的决策机制，制定出台市人民政府重大决策合法性审查规定，促进依法决策、科学决策、民主决策。全面落实民族区域自治制度，深入贯彻“一法两规定”，不断巩固和发展平等团结互助和谐的社会主义民族关系。继续落实“五五”普法规划。

加强应急管理体系建设，切实提高预防和处置突发公共事件的能力。整合各方资源，加快应急平台建设，努力形成政府主导、部门协作、军地结合、全社会共同参与的应急管理工作格局。建立健全重大突发公共事件新闻报道快速反应机制、舆情收集和分析机制，切实提高预防和处置突发公共事件的能力。

加强党风廉政建设，切实做到为民、务实、清廉。认真落实党风廉政建设责任制，抓好领导干部廉洁自律工作，进一步加大查办案件工作力度，坚决纠正损害群众利益的不正之风。加强对中央新增投资项目和重点项目实施的监管，主动接受人民群众和社会各界的监督，绝不允许在项目实施过程中为单位和个人谋取私利。牢固树立过“紧日子”的思想，继续发扬艰苦奋斗、勤俭节约的作风，坚决反对铺张浪费，真正把有限的财力用在“刀刃”上。

各位代表，安顺的发展正处在一个关键时期，困难和挑战考验着我们，责任和使命激励着我们。我们坚信，任何困难都动摇不了全市人民开创美好生活的坚定信念，任何挑战都阻挡不了我们前进的步伐。让我们更加紧密地团结在以胡锦涛同志为总书记的党中央周围，高举中国特色社会主义伟大旗帜，以邓小平理论和“三个代表”重要思想为指导，全面深入贯彻落实科学发展观，在中共安顺市委的坚强领导下，在市人大、市政协的监督和支持下，坚定信心、抢抓机遇，同心同德、共克时艰，开拓创新、奋力拼搏，把富民兴安宏伟大业不断推向前进，以优异成绩向新中国成立60周年献礼！
